# Triglyceride to HDL-C ratio is associated with plasma D-dimer levels in different types of pancreatitis

**DOI:** 10.1038/s41598-022-17421-7

**Published:** 2022-07-28

**Authors:** Xiaoqing Jia, Xiaoting Zhang, Dalong Sun, Na Yang, Rong Li, Zheng Luo

**Affiliations:** 1 Department of Gastroenterology and Hepatology, Qilu Hospital of Shandong University, 107 West Wenhua Road, Jinan, 250012 Shandong China; 2 Department of Geriatric Medicine, Qilu Hospital, Shandong University, 107 West Wenhua Road, Jinan, 250012 Shandong China

**Keywords:** Diseases, Risk factors, Signs and symptoms

## Abstract

This study aims to evaluate levels of D-dimer and serum lipid in different types of pancreatitis, and the relationship between D-dimer and dyslipidemia, especially triglyceride to HDL-C ratio (TG/HDL-C) in different types of pancreatitis. We analyzed the D-dimer and dyslipidemia levels in acute pancreatitis (AP), recurrent acute pancreatitis (RAP) and chronic pancreatitis (CP). A single-centered retrospective study was conducted on 1013 patients diagnosed with AP, RAP or CP. Only patients hospitalized within 24 h of onset were included, and 204 patients were enrolled in pancreatitis groups. 68 normal persons without pancreatitis, malignant diseases, pregnancy, or organ failure, who had health check-ups, were enrolled in the control group. Blood samples were taken within 24 h of admission. The relevant information on epidemiology and etiology was collected. D-dimer and serum lipid levels in different types of pancreatitis were analyzed. Furthermore, the area under the receiver-operating characteristic curve (AUC) was used to estimate the validity of the predictor and to define optimal cut-off points for prediction. We found that D-dimer and TG/HDL-C ratio could distinguish mild AP (MAP) and non-MAP in AP and RAP patients. The D-dimer level was related to TG/HDL-C ratio and severity of pancreatitis, with the coefficient correlation of 0.379 and 0.427(p < 0.05), respectively. TG/HDL-C was related to D-dimer in different types of pancreatitis. Logistic regression analysis was conducted in the parameters at admission like alcohol abuse, dyslipidemia and coagulation disturbance in distinguishing AP and RAP groups from the control group, and the parameter like diabetes in RAP and CP groups significantly increased compared with that of the control group. The value of D-dimer level and TG/HDL-C ratio in predicting the severity of AP and RAP was confirmed but there was no significant difference between CP group and the control group. The D-dimer level was related to dyslipidemia and TG/HDL-C ratio.

## Introduction

Acute pancreatitis (AP) occurs due to the autodigestion of the pancreatic enzymes. It is an acute inflammatory process characterized by severe pain localized in the upper abdomen which may be radiating towards the back and can trigger a systemic inflammatory response^1^. Recurrent acute pancreatitis (RAP) is a clinical condition characterized by repeated episodes of acute pancreatitis, which is diagnosed retrospectively by clinical definition after at least the second episode of AP^2^. RAP is an intermediary stage in the pathogenesis of chronic pancreatitis (CP), and a subset of RAP patients during their natural course transition to CP^3^. CP involves progressive inflammatory and fibrotic changes of the exocrine pancreas, which could result in permanent structural damage and lead to impairment of both endocrine and exocrine functions^4^. It is known that the socioeconomic burden of pancreatitis is significant given the presence of symptoms and the costs to the health system^5^. The severe form accounting for 20–30% of acute pancreatitis patients is a life-threatening disease with high nosocomial mortality^6^. Therefore, identifying risk factors for RAP in patients with AP as early as possible may improve prevention and early treatment, thereby improving prognosis.

Pancreatitis induces venous thrombosis^7–9^. The pathogenesis of pancreatitis is release and activation of numerous proinflammatory cytokines leading to hypercoagulation and microvascular thrombosis, which may result in multi-organ dysfunction, splenic thrombosis and venous thromboembolism^10–14^. Thrombosis is a vascular complication of pancreatitis and a major cause of morbidity and mortality. Chronic pancreatitis accompanied by isolated thrombosis of the splenic vein is caused by perivenous inflammation of RAP^15^.

D-dimer is the cleavage product of cross-linked fibrin that is formed by activation of the coagulation system, which signals hyperfibrinolysis in response to clot activation and fibrin formation^16^. D-dimer assays are commonly used in clinical practice to diagnose deep vein thrombosis or pulmonary embolism^17^. Recently, D-dimer levels are elevated in a variety of conditions, including coronary artery disease, infection and atrial fibrillation^18–20^. D-dimer is also found to be an early predictor of severity of acute pancreatitis^13^. However, the correlation between D-dimer levels and different types of pancreatitis is relatively unexplored.

Dyslipidemia include a low level of high-density lipoprotein cholesterol (HDL-C) and increased level of triglyceride (TG). The triglyceride to HDL-C ratio (TG/HDL-C) summarizes the combined effect of each level and can be used as a better predictor for insulin resistance, cardio-metabolic risk, and cardiovascular disease^21–23^. Besides, Several studies have reported that an elevated TG/HDL-C ratio correlated with the prevalence of CKD^24^. To our knowledge, few studies have evaluated TG/HDL-C ratios in different types of pancreatitis, and the relationship between TG/HDL-C and D-dimer has not been investigated.

In the present study, the plasma levels of D-dimer in patients with AP, RAP and CP were measured. Subsequently, correlations between D-dimer levels and different types of pancreatitis were assessed.

## Results

### Patient and control characteristics

**Figure 1:**
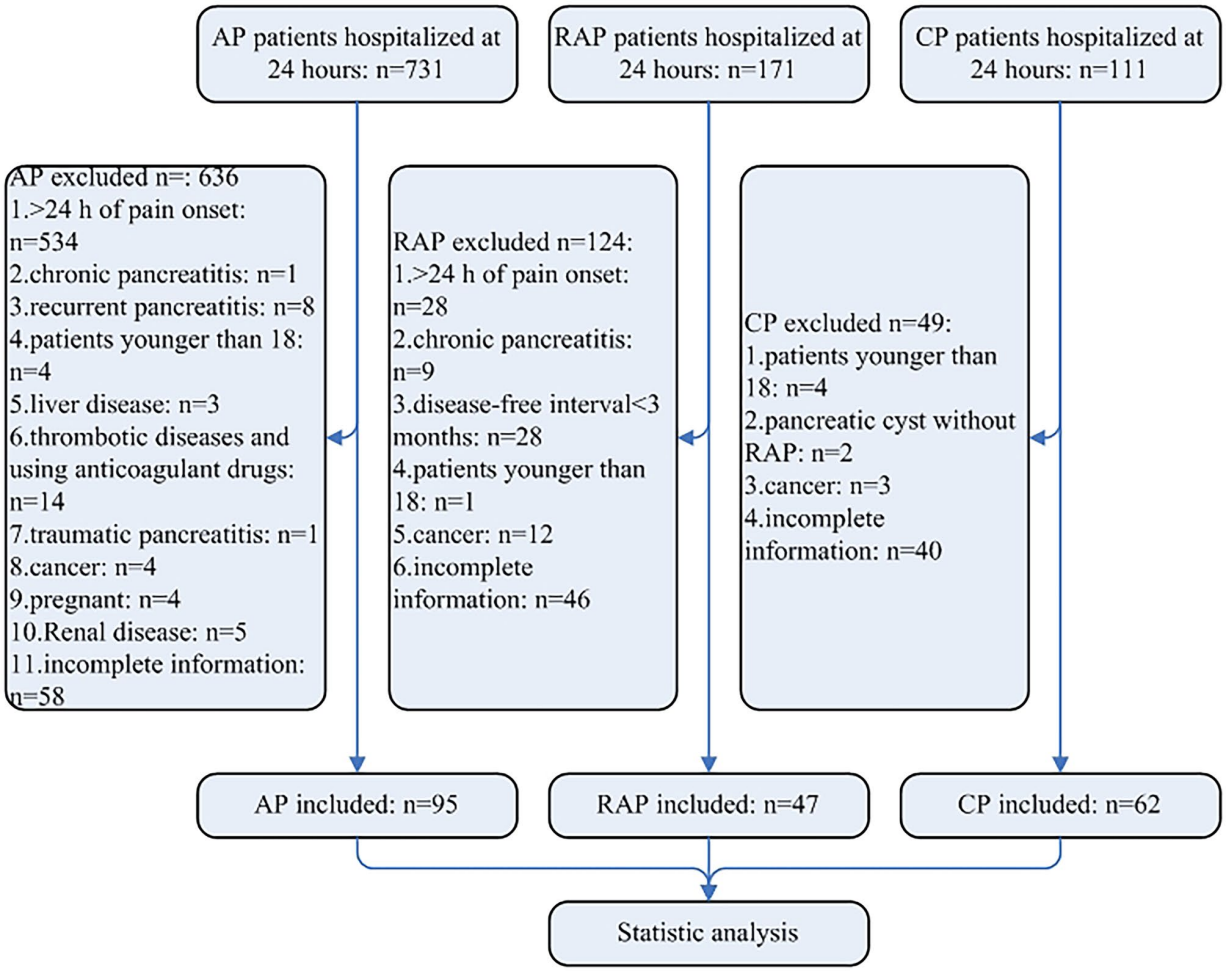
Flow chart of the study.

Of 1013 patients with pancreatitis, 204 patients were enrolled and their serum D-dimer levels were all measured within 24 h of hospital admission. Baseline demographic and clinical characteristics were analyzed toward 95 patients with AP, 47 patients with RAP and 62 patients with CP (Fig. 1). Besides, 68 patients without pancreatitis, malignant diseases, pregnancy, or organ failure, who had regular physical check-ups, were enrolled in the control group. Of the 95 patients with AP, 40.00% were female and 60.00% were male, the median age was 50. In the RAP group, 27.66% were female and 72.34% were male, the median age was 45. Of the patients with CP, females were 20.97%, and males were 79.03%, the median age was 52.5. In the control group, 33.82% were female and 66.18% were male, with a median age of 51 (Table 1). In AP patients, 35.79% had a smoking history, 41.05% had a history of drinking alcohol, 32.63% had hypertension, 9.47% had a history of cardiovascular disease (CVD), 13.68% had type 2 diabetes, 20.00% had allergy history, 5.26% had family chronic diseases, 23.16% had dyslipidemia and 70.53% had coagulation disorder. In RAP patients, 44.68% had a smoking history, 38.30% had a history of drinking alcohol, 14.89% had hypertension, 17.02% had a history of cardiovascular disease (CVD), 25.53% had type 2 diabetes, 17.02% had allergy history, 8.51% had family chronic diseases, 19.15% had dyslipidemia and 57.45% had coagulation disorder. In CP patients, 61.29% had a smoking history, 67.74% had a history of drinking alcohol, 30.65% had hypertension, 8.06% had a history of cardiovascular disease (CVD), 29.03% had type 2 diabetes, 9.68% had allergy history, 8.06% had family chronic diseases, 1.61% had dyslipidemia and 25.81% had coagulation disorder. Also, common etiologies, including biliary dysfunction, hypertriglyceridemia, alcoholism and idiopathic pancreatitis in different types of pancreatitis were shown in Table 1.

**Table 1:** Patients’ characteristics and predictors by univariate analysis in different types of pancreatitis.

Variable	AP	RAP	CP	Control
**Gender**				
Female (N%)	40.00%	27.66%	20.97%	33.82%
Male (N%)	60.00%	72.34%	79.03%	66.18%
OR(95%CI)	0.77 (0.40–1.47)	1.35 (0.67–2.72)	1.93 (0.87–4.25)	
**Age**				
Median age	50 (18–91)	45 (22–94)	52.5 (30–78)	51 (18–79)
OR (95%CI)	1.00 (0.97–1.03)	1.00 (0.98–1.02)	0.97 (0.93–1.01)	
**Smoking**				
No (N%)	64.21%	55.32%	38.71%	77.94%
Yes (N%)	35.79%	44.68%	61.29%	22.06%
OR	0.98 (0.24–4.10)	2.71 (0.39–18.85)	1.07 (0.18–6.47)	
**Alcoholic abuse**				
No (N%)	58.95%	61.70%	32.26%	86.76%
Yes (N%)	41.05%	38.30%	67.74%	13.24%
OR(95%CI)	5.98 (1.48–24.15)*	9.12 (1.31–63.6)*	26.65 (4.39–161.67)**	
**Hypertension**				
No (N%)	67.37%	85.11%	69.35%	80.88%
Yes (N%)	32.63%	14.89%	30.65%	19.12%
OR(95%CI)	1.70 (0.50–5.72)	0.74 (0.10–5.15)	1.35 (0.32–5.74)	
**Heart disease**				
No (N%)	90.53%	82.98%	91.94%	95.59%
Yes (N%)	9.47%	17.02%	8.06%	4.41%
OR(95%CI)	4.88(0.61–39.26)	16.64 (0.58–475.29)	10.64 (1–113.62)	
**Diabetes**				
No (N%)	86.32%	74.47%	70.97%	92.65%
Yes (N%)	13.68%	25.53%	29.03%	7.35%
OR (95%CI)	0.41 (0.07–2.40)	2.67 (0.29–24.65)	7.36 (1.28–42.23)*	
**Allergic history**				
No (N%)	80.00%	82.98%	90.32%	89.71%
Yes (N%)	20.00%	17.02%	9.68%	10.29%
OR(95%CI)	2.95 (0.71–12.23)	2.22 (0.35–13.89)	0.90 (0.15–5.25)	
**Family chronic disease**				
No (N%)	94.74%	91.49%	91.94%	98.53%
Yes (N%)	5.26%	8.51%	8.06%	1.47%
OR (95%CI)	6.27 (0.05–794.39)	1.88E + 08 (0–)	4.1E + 07 (0–)	
**Dyslipidemia**				
No (N%)	76.84%	80.85%	98.39%	75.00%
Yes (N%)	23.16%	19.15%	1.61%	25.00%
OR(95%CI)	8.40 (4.11–17.15)**	10.24 (4.74–22.11)**	2.09 (0.63–6.91)	
**Coagulation disorder**				
No (N%)	29.47%	42.55%	74.19%	85.29%
Yes (N%)	70.53%	57.45%	25.81%	14.71%
OR (95%CI)	13.88 (6.22–30.98)**	12.53 (5.51–28.49)**	3.43 (0.63–6.91)	
**Etiology**				
Alcoholic	15.79%	14.89%	32.26%	
Biliary	64.21%	34.04%	53.23%	
Hypertriglyceridemia	5.26%	17.02%	0.00%	
Idiopathic	14.74%	10.64%	14.52%	
**Severity**				
MAP	86.32%	93.62%		
MSAP	8.42%	4.26%		
SAP	5.26%	2.13%		

*AP* acute pancreatitis, *RAP* recurrent acute pancreatitis, *CP* chronic pancreatitis.

*p < 0.05; **p < 0.01.

Gender, age, smoking status, alcohol drinking status, allergic history, presence of CVD, hypertension, type 2 diabetes, dyslipidemia, and coagulation disorder of these patients had been undergone logistic regression analysis.

AP patients with coagulation disorder had an odds ratio (OR) of 13.88 (95% CI 6.22–30.98; P = 0.00) compared with patients in control group (Table 1). RAP patients with coagulation disorder had an OR of 12.53 (95% CI 5.51–28.49; P = 0.00) compared with patients in control group. AP patients with dyslipidemia had an OR of 8.40 (95% CI 4.11–17.15; P = 0.00) compared with patients in control group. RAP patients with dyslipidemia had an OR of 10.24 (95% CI 4.74–22.11; P = 0.00) compared with patients in control group. CP patients with dyslipidemia had an OR of 2.46 (95% CI 1.16–5.19; P = 0.02). CP patients with coagulation disorder had no significant difference compared with control group. AP patients with alcohol abuse had an OR of 6.74 (95% CI 2.18–20.85; P = 0.00) compared with patients in control group. RAP patients with alcohol abuse had an OR of 7.41 (95% CI 1.8–30.55; P = 0.01) compared with patients in control group. CP patients with alcohol abuse had an OR of 18.89 (95% CI 3.84–93; P = 0.00) compared with patients in control group. RAP patients with type 2 diabetes had an OR of 7.73 (95% CI 2.08–28.69; P = 0.00) compared with patients in control group. CP patients with type 2 diabetes had an OR of 6.71 (95% CI 1.73–26.06; P = 0.01) compared with patients in control group, besides AP patients with type 2 diabetes had no significant difference compared with control group. RAP patients with heart disease had an OR of 10.15 (95% CI 1.91–53.82; P = 0.01) compared with patients in control group. There was no significant association in age, gender, smoking, hypertension, family chronic disease and allergic history between patients with different types of pancreatitis and the control group.

### D-dimer, dyslipidemia and TG/HDL-C ratios in different types of pancreatitis

**Figure 2:**
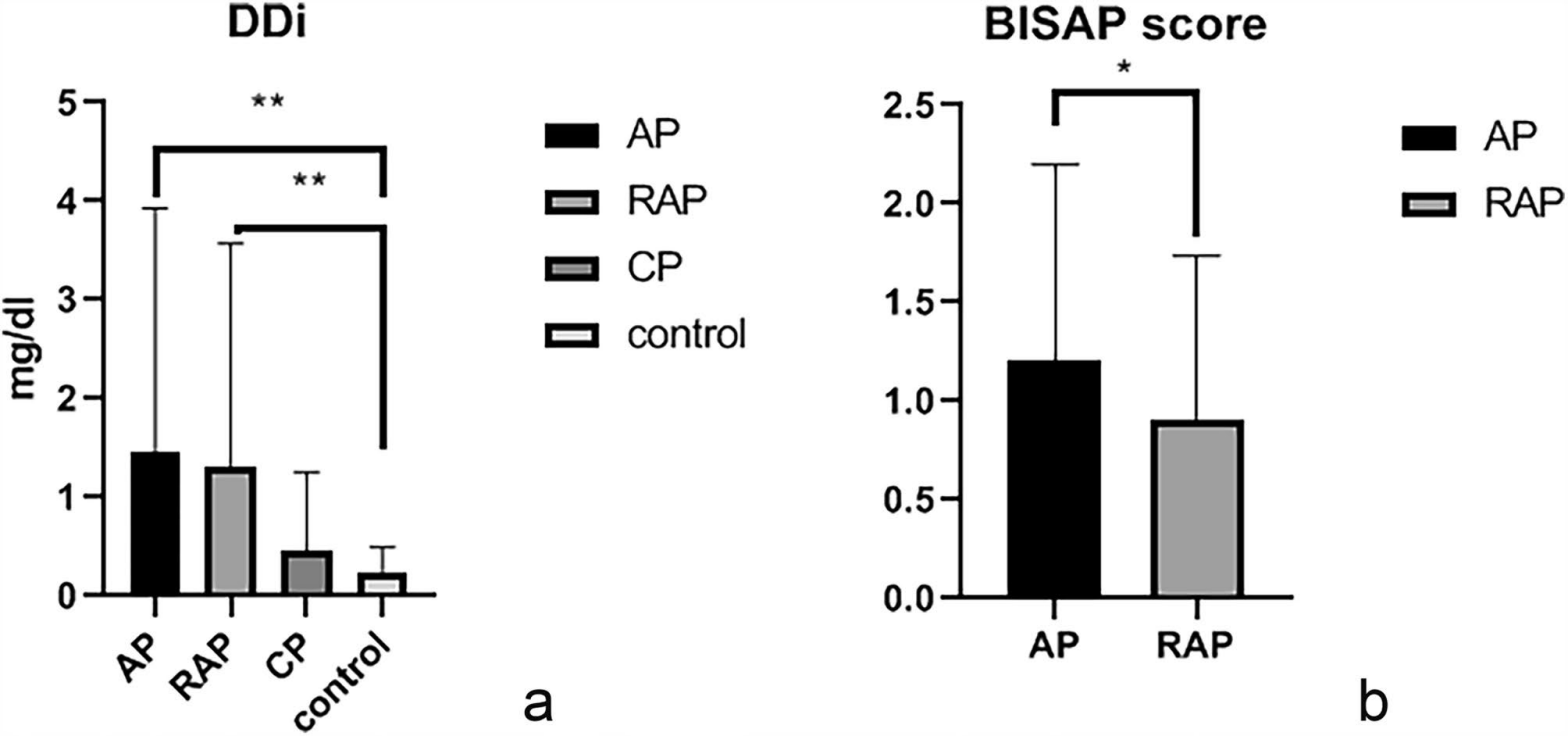
D-dimer level in different types of pancreatitis and severity of different types of pancreatitis: (**a**) D-dimer expression in types of pancreatitis. (**b**) BISAP score in different types of pancreatitis. *p < 0.05; **p < 0.01.

In different types of pancreatitis, D-dimer levels in AP patients and RAP patients were significantly higher than those in control group (p < 0.05), and there was no significant difference in D-dimer levels between CP group and control group (Table 2, Fig. 2). AP and RAP patients were subdivided into MAP and non-MAP groups (MSAP and SAP groups). For the prediction of severity of AP, the area under curve (AUC) of non-MAP group for serum D-dimer levels was 0.851 (p < 0.05), the cut-off value was 1.13 mg/dL. For the prediction of RAP severity, the AUC of non-MAP group for serum D-dimer levels was 0.80 (p < 0.05), the cut-off value was 2.14 mg/dL (Table 3, Fig. 4).

**Table 2:** Dyslipidemia and severity in types of pancreatitis.

		AP (mean ± SD)	RAP (mean ± SD)	CP (mean ± SD	Control (mean ± SD)	DDi corelation coefficient
Dyslipidemia	Cho (mmol/L)	4.49 ± 2.10	4.84 ± 1.62	4.29 ± 2.37	4.35 ± 0.81	− 0.12
	TG (mmol/L)	2.23 ± 2.36**	2.58 ± 2.20**	1.62 ± 1.31	1.21 ± 0.49	0.141*
	HDL-C (mmol/L)	0.9 ± 0.42**	0.97 ± 0.42**	1.04 ± 0.32**	1.25 ± 0.25	− 0.35
	LDL-C (mmol/L)	2.59 ± 0.96	2.69 ± 1.06	2.65 ± 2.19	2.59 ± 0.63	− 0.14
	NEFA (umol/dl)	61.07 ± 40.3**	50.97 ± 24.12*	48.37 ± 28.33	39.09 ± 24.94	0.146*
	TG/HDL-C	3.08 ± 3.09**	4.74 ± 10.86*	1.73 ± 1.76*	1.05 ± 0.73	0.379**
BISAP score		1.29 ± 0.11*	0.96 ± 0.1			0.427**
DDi(mg/dl)		1.46 ± 2.47**	1.32 ± 2.25**	0.46 ± 0.80	0.24 ± 0.26	

*AP* acute pancreatitis, *RAP* recurrent acute pancreatitis, *CP* chronic pancreatitis, *TG* triglyceride, *Cho* cholesterol, *LDL-C* low-density lipoprotein cholesterol, *HDL-C* high-density lipoprotein cholesterol, *NEFA* non-esterified fatty acids, *BISAP* bedside index of severity in acute pancreatitis.

*p < 0.05; **p < 0.01.

**Table 3:** ROC cure and cut-off value of D-dimer and dyslipidemia in different types of pancreatitis.

DDi vs severity of pancreatitis^#^		AP			RAP			CP		
		AUC	Cut-off vaue	p	AUC	Cut-off vaue	p	AUC	Cut-off vaue	p
Dyslipidemia vs severity of pancreatitis		0.85	1.13	0.00	0.80	2.14	0.04			
	Cho	0.17	–	0.00	0.21	2.22	0.05			
	TG	0.50	1.39	0.97	0.63	2.09	0.39			
	HDL-C	0.25	–	0.00	0.08	0.22	0.00			
	LDL-C	0.14	4.05	0.00	0.34	1.42	0.29			
	NEFA	0.46	237.00	0.65	0.28	10.50	0.14			
	TG/HDL-C	0.61	2.17	0.22	0.84	3.51	0.02			
Dyslipidemia vs Ddi	Cho	0.36	6.42	0.02	0.50	5.98	0.98	0.46	3.33	0.65
	TG	0.61	1.37	0.08	0.70	2.17	0.00	0.58	1.18	0.37
	HDL-C	0.26	2.39	0.00	0.36	1.49	0.03	0.23	–	0.00
	LDL-C	0.36	3.86	0.02	0.48	3.52	0.74	0.52	1.86	0.83
	NEFA	0.45	40.50	0.46	0.55	42.50	0.44	0.75	46.50	0.00
	TG/HDL-C	0.69	2.56	0.00	0.73	2.10	0.00	0.67	2.31	0.05

*AP* acute pancreatitis, *RAP* recurrent acute pancreatitis, *CP* chronic pancreatitis, *TG* triglyceride, *Cho* cholesterol, *LDL-C* low-density lipoprotein cholesterol, *HDL-C* high-density lipoprotein cholesterol, *NEFA* non-esterified fatty acids, *AUC* area under the receiver-operating characteristic curve.

^#^The severity of pancreatitis was subdivided into MAP and non-MAP groups.

**Figure 3:**
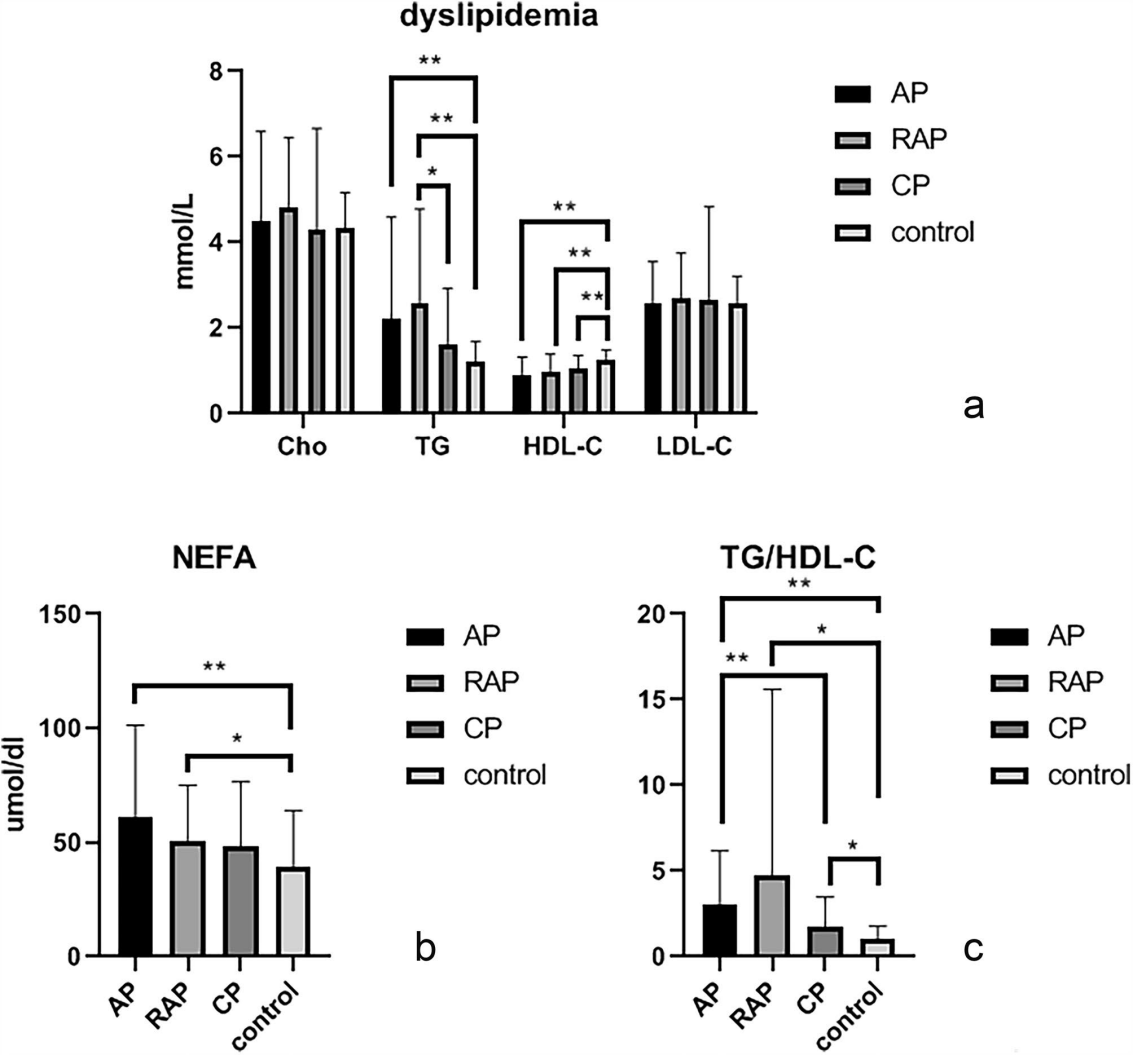
Dyslipidemia in different types of pancreatitis. (**a**) Dyslipidemia in different types of pancreatitis; (**b**) Free fatty acid in different types of pancreatitis; (**c**) TG/HDL-C ratio in different types of pancreatitis. *p < 0.05; **p < 0.01.

**Figure 4:**
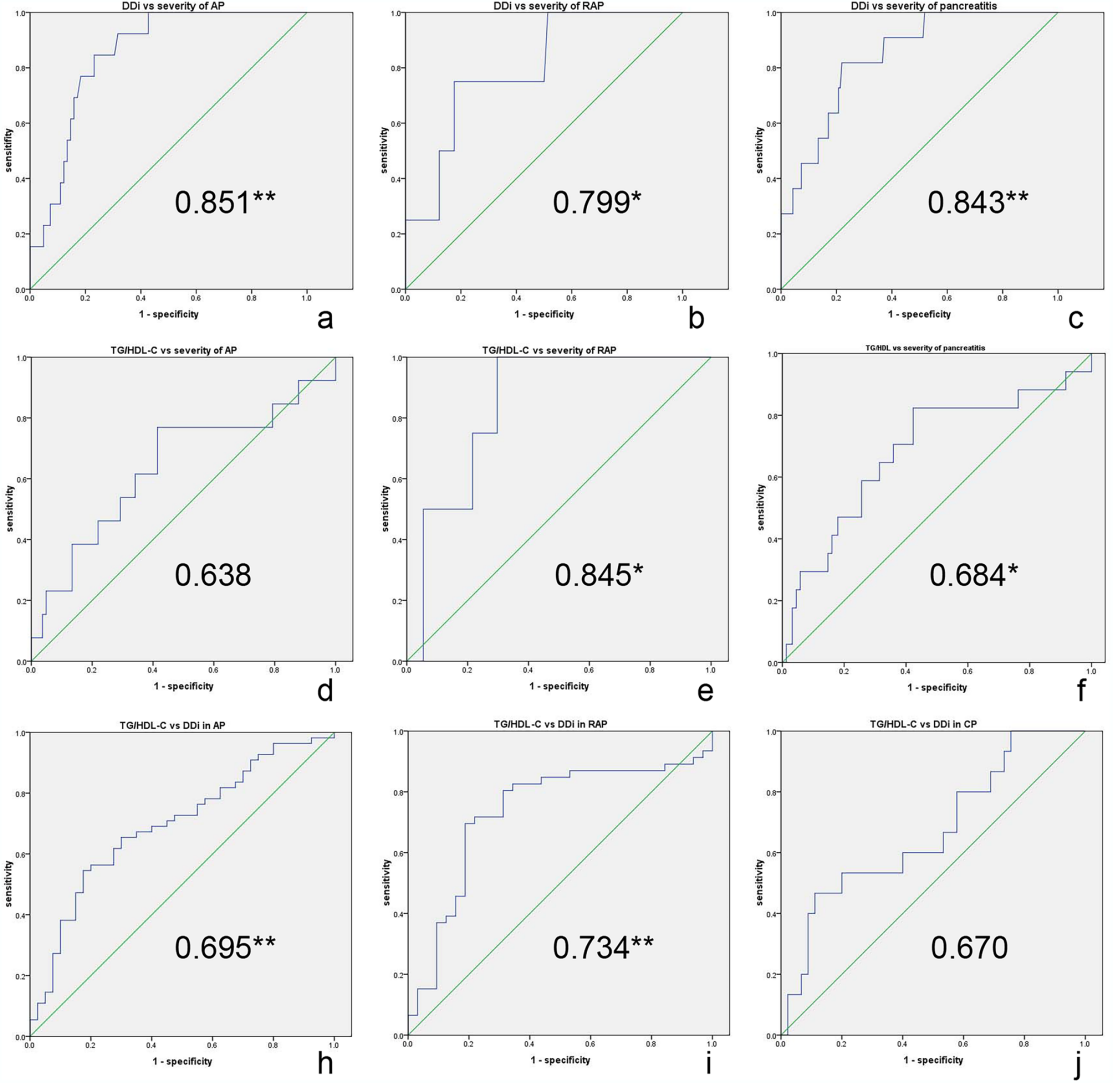
ROC curves of D-dimer and dyslipidemia in different types of pancreatitis. (**a**) ROC curves for D-dimer level to distinguish MAP and non-MAP group in AP. (**b**) ROC curves for D-dimer level to distinguish MAP and non-MAP group in RAP. (**c**) ROC curves for D-dimer level to distinguish MAP and non-MAP group. (**d**) ROC curves for TG/HDL-C level to distinguish MAP and non-MAP group in AP. (**e**) ROC curves for TG/HDL-C level to distinguish MAP and non-MAP group in RAP. (**f**) ROC curves for TG/HDL-C level to distinguish MAP and non-MAP group. (**g**) ROC curves for TG/HDL-C level to distinguish normal D-dimer group and increased D-dimer group in AP. (**h**) ROC curves for TG/HDL-C level to distinguish normal D-dimer group and increased D-dimer group in RAP. (**i**) ROC curves for TG/HDL-C level to distinguish normal D-dimer group and increased D-dimer group in CP. *p < 0.05; **p < 0.01.

In this study, the severities of pancreatitis were recognized according to 2012 Atlanta classification, and measured by BISAP score^25^, the BISAP scores of AP patients were significantly higher than those of RAP group (p < 0.05). The correlation coefficient between the BISAP score and D-dimer level was 0.427 (p < 0.05) (Table 2, Fig. 2). Our studies showed that in different types of pancreatitis, the dyslipidemia was significantly different between the AP, RAP group and the control group. Therefore, dyslipidemia in each group was analyzed. Levels of HDL-C in different types of pancreatitis were significantly lower than those in control group but had no significant difference between each pancreatic group. Levels of TG and non-esterified fatty acid (NEFA) were significantly higher in AP and RAP groups than those in control group (p < 0.05) but were not significantly different between AP and RAP groups. TG/HDL-C ratios in different types of pancreatitis were significantly higher than those in control group (Table 2, Fig. 3). The relationship between D-dimer level and dyslipidemia occurrence was analyzed, and the correlation coefficient between them was 0.33 (p < 0.05) (Table 2). For the prediction of RAP severity, AUC of non-MAP group for TG/HDL-C ratio was 0.84 (p < 0.05), the cut-off value was 3.51 (Table 3, Fig. 4). For the prediction of the relationship between TG/HDL-C and D-dimer level in pancreatitis, the patients were divided into D-dimer elevated group and normal D-dimer group, the AUC of D-dimer elevated group for TG/HDL-C ratio was 0.69 (p < 0.05), the cut-off value was 2.56 (p < 0.05), in RAP the AUC was 0.73, and the cut-off value was 2.10, but there is no significant relationship between D-dimer and TG/HDL-C ratio in CP group (Table 3, Fig. 4).

## Discussion

### Characters of patients with different types of pancreatitis

RAP refers to a clinical entity characterized by episodes of acute pancreatitis which occurs on more than one occasion. An established chronic disease may be found either on the occasion of the first episode of pancreatitis or during the follow-up. RAP was the strongest predicting factor for a subsequent diagnosis of CP^4,26,27^. The etiology and epidemiology of AP, RAP and CP were studied in this research. The results showed that coagulation disorder is related to AP and RAP group, with OR of13.88 and 12.53 (P < 0.05) respectively compared with patients in control group, and D-dimer levels in AP and RAP groups were significantly higher than those of the control group. However, coagulation disorder and D-dimer levels had no significant difference between CP group and control group. The D-dimer level is also related to the severity of pancreatitis. This result was confirmed in other studies, which showed that D-dimer was an early predictor of the severity of AP^28–30^. The possible mechanism may be two-sided: the abnormal activation of pancreatic enzymes results in inflammation and injury to the pancreas at the onset of AP, which then induces thrombosis and further aggravates the injury^31,32^. The systemic inflammatory response syndrome and multiple organ dysfunction syndromes, which occur in patients with AP, are common risk factors for the development of VTE^33^. The propagation of the acute inflammatory response can lead to chronic inflammation if there is no appropriate resolution^34,35^. On a molecular level, pancreatic stellate cells (PaSCs) have been found to play an important role in models of CP. In CP, PaSCs participate in pathogenesis after transforming into an activated or “myofibroblastic” state^36^. In this myofibroblastic state, PaSCs produce collagen and other extracellular matrix proteins that lead to fibrosis. PaSCs also secrete cytokines which further promote the inflammatory process^36^. However, the D-dimer levels of CP patients were not very different from those of the control group; the reason may be that the severity of inflammation in CP patients was less than that of AP and RAP groups. Besides, the fibrotic changes of the pancreas may not induce acute inflammation and coagulation disturbance. A study about splanchnic venous thrombosis (SVT) suggested that local inflammation plays a major role in SVT causation. Thrombophilia caused by coagulation disturbance is seen in one-third of patients with AP but does not seem to increase the risk of SVT^37^.

Our study shows that dyslipidemia is related to different types of AP, RAP and CP, with OR of 8.40, 10.24 and 2.46 (p < 0.05) respectively, and dyslipidemia is also related to D-Dimer level. In previous studies, dyslipidemia, especially hypertriglyceridemia, is related to AP and RAP^38^, the pathophysiology could be the metabolism of excessive TGs by pancreatic lipase to non-esterified fatty acids (NEFA) leading to pancreatic cell injury and ischemia^39^. A 3558-patient study comparing high triglyceride-AP (HTG-AP) versus non-HTG-AP s reported a statistically significant higher incidence of pancreatic necrosis, infected pancreatic necrosis, organ failure, and persistent organ failure^40^. Recent studies demonstrate a trend towards severity in patients with HTG-AP when compared with non-HTG pancreatitis patients; the patients with higher TGs levels appear to have more severe hospital courses with a higher incidence of complications (35–69%) and organ failure (20–35%)^41^. Researches have shown that patients with diabetes, obesity, or metabolic syndrome due to insulin resistance tend to have low HDL-C because of lower lipoprotein lipase activity and triglyceride enrichment, but the mechanism is still unknown^42–44^. The TG/HDL-C ratio was found to be associated with insulin resistance in overweight and obese children^45^. Our research shows that in AP and RAP patients, TG and NEFA levels of which were significantly higher than those of CP and control group, HDL-C levels of AP, RAP and CP groups were lower than those of the control group (p < 0.05), TG/HDL-C ratios of AP, RAP and CP groups were significantly higher than those of the control group (p < 0.05), which may be related to insulin resistance in all types of pancreatitis^46,47^. TG/HDL-C ratio is also a potential useful marker to identify the severity of RAP patients in clinical practice, with positive predictive value of 0.84 at 3.51 cut-off point. TG/HDL-C ratio was related to insulin resistance, which may occur after acute pancreatitis^46^. In our research, TG/HDL-C ratio is found to be a predictor of severity in RAP patients but not in first attack of AP. The mechanism may be that hypertriglyceridemia (HTG) is a well-established cause of RAP, and TG/HDL-C ratio is predictable in RAP. The relationship between insulin resistance and RAP should be further investigated^39^.

We also found that D-dimer level was related to TG/HDL-C ratio (r = 0.379, p < 0.05). A previous study has shown that both TG and acute pancreatitis could cause an elevation in d-dimer level, in which TG plays a more important role^48^. In type 2 diabetic children and adolescents, D-dimer level was significantly correlated with TG (p < 0.05)^49^. Our research showed that TG was related to D-dimer level in RAP group, the AUC of TG in elevated D-dimer group were less than AUC of TG/HDL-C ratio, the correlation coefficient between TG and D-dimer is less than TG/HDL-C ratio and D-dimer level. So TG/HDL-C could be used as a better predictor for RAP severity and elevated D-dimer level.

Alcoholism is also a risk factor of all types of pancreatitis. Recent data from predominant western countries have shown an increasing trend in the incidence of acute pancreatitis and the number of hospital admissions for both acute and chronic pancreatitis^50–52^. The pathogenesis may be PSCs, which are activated directly by alcohol and its metabolites, and also by cytokines and growth factors released during alcohol-induced pancreatic necroinflammation. Activated PSCs are the key cells responsible for fibrosis of alcoholic chronic pancreatitis^53^. Our research shows that alcoholism is related to AP, RAP and CP groups, with OR of 6.74, 7.41 and 18.89 (p < 0.05) respectively.

Our research shows that diabetes is related to RAP and CP. The pathogenesis is that RAP and CP, which are pancreatic inflammation with irreversible parenchymal damage and functional changes, is complicated by progressive nutrient maldigestion, glucose intolerance, diabetes mellitus, and metabolic derangements^54^. Destruction of islet cells by pancreatic inflammation can lead to the development of “brittle” disease with wide swings in blood sugar which are difficult to control. Besides, patients may have pre-existing risk factors for type 2 diabetes, such as insulin resistance, obesity, or dietary habits that further complicate the optimal regulation of glucose metabolism^55–57^. In our study, diabetes was related to RAP and CP groups, with OR of 7.73 and 6.71 (p < 0.05), but had no relationship to the AP group.

In our study, heart disease is related to RAP, with OR of 10.15 (p < 0.05). So far, research on association between heart and pancreas disease has been paid little attention and its role in pathogenesis is not fully elucidated. The pathogenesis could be pancreatic enzymes and their inhibitors that profoundly affected blood coagulation and appear to influence the course of pancreatic inflammation^58^. Researches have shown that in patients with chronic heart failure, the splanchnic circulation is decreased, especially in highly vascularized pancreas, which may cause pancreatitis^59^.

There are no significant differences in other factors like smoking, hypertension, etc. between pancreatitis and the control group. Although we drew similar conclusions from recent studies, our research had significant advantages. We distinguished different types of pancreatitis between AP, RAP and CP groups. RAP is a syndrome of multiple distinct acute inflammatory responses originating within the pancreas in individuals with genetic, environmental, traumatic, morphologic, metabolic, biologic, and/or other risk factors who experienced 2 or more episodes of documented AP, and RAP may lead to chronic pancreatitis^60^. Approximately 9% to 31% of patients with AP develop RAP^61,62^. To our knowledge, no similar investigations about a comparison between AP, RAP and CP groups have been investigated in China. In our research, the epidemiology and etiological factors of RAP are similar to those of first attack AP; also D-Dimer levels and dyslipidemia were similar in the RAP and the AP groups. However, the D-Dimer level, dyslipidemia and severity of pancreatitis in CP group were similar to those of the control group.

The present research has several limitations. Firstly, it was conducted in a single center with a relatively small sample, which may create bias. Secondly, we did not take into account some inflammation factors, such as PCT, C-reactive protein and interleukin-6; or arterial blood gases, which are also useful predictors of severity in AP^63,64^, because they were not routinely analyzed at admission in Qilu Hospital. Besides, we used BISAP score instead of APACH-II score to analyze the severity of pancreatitis because some test results needed in the APACH-II score system were not available, and research had confirmed that BISAP is as useful as APACHE-II and more effective than Ranson criteria, CTSI, CRP, HCT and BMI in predicting severity, organ failure, and death in patients with acute pancreatitis^25^. However, it may still create bias, especially when compared with other studies.

## Conclusion

We confirmed the diagnostic value of D-dimer and TG/HDL-C ratio in predicting the severity of AP and RAP. This suggested that D-dimer might be a useful early predictive biomarker for non-MAP of AP and RAP groups, and TG/HDL-C ratio could be a predictive index for non-MAP of RAP patients, but as the disease progress to CP, the value of D-dimer level and TG/HDL-C ratio decreases. The TG/HDL-C ratio is related to D-dimer level in AP and RAP groups. These simple and feasible marker and index could be used in clinical practice to improve early management of AP and RAP, which suggests that the early intervention of coagulation disturbance and dyslipidemia could be substantial. The relationship between D-dimer and dyslipidemia need further investigation.

## Material and methods

In this observational study, the data of all patients were retrospectively collected from Shandong University Qilu hospital from January 1, 2017 to December 31, 2020. The ethics committee of Qilu Hospital of Shandong University reviewed and approved this study (No.KYLL-202008-096). All the data are anonymous, and the requirement for informed consent was therefore waived. The informed consent waiver statement was approved by the ethics committee of Qilu Hospital of Shandong University. The approval was supported in Supplementary Material.

### Study population

A review of all medical registries with International Disease Classification-10 diagnoses related to AP and CP from January 2017 to December 2020 in Shandong University Qilu hospital was performed; RAP patients were selected from AP patients on the basis of RAP definition.

According to the Atlanta classification and definition of acute pancreatitis, AP was defined as the presence of two or more of the following: (a) abdominal pain compatible with AP, (b) serum amylase and/or lipase values ≥ 3 times upper limits of normal, (c) imaging findings of AP^65^. RAP was defined as 2 or more well-documented separate attacks of AP with complete resolution for 3 or more months between attacks according to current definition of RAP^66^. CP patients were selected from patients with CP diagnosis and with typical features of epigastric pain, steatorrhea, weight loss, or recurrent acute pancreatitis, and CT or MRI results which were considered consistent with chronic pancreatitis according to the American Pancreatic Association Practice Guidelines^67^. Normal persons without pancreatitis, malignant diseases, pregnancy, or organ failure, who had health check-ups were enrolled in the control group.

The exclusion criteria were as follows and the flow chart was shown in Fig. 1:

- Participants who failed to meet the definition of AP, RAP or CP were excluded in each group.
- In AP and RAP groups, variables obtained from laboratory exams over the first 24 h of the onset of symptoms were excluded.
- Participants younger than 18 or pregnant participants were excluded.
- Participants with pre-existing chronic diseases like malignant tumor, heart diseases, liver diseases and renal diseases which may influence the variables were excluded. Participants with traumatic pancreatitis were also excluded in present study.
- Participants with incomplete information were excluded.

The severities of AP and RAP patients were well recognized according to the latest 2012 revision of the Atlanta classification. Mild AP (MAP) patients were not associated with organ failure (OF) and local or systemic complications. Moderately severe AP (MSAP) was characterized by the presence of transient OF (less than 48 h) or local or systemic complications. Severe AP (SAP) was defined as persistent OF for more than 48 h. The diagnosis of OF was based on the modified Marshall scoring system, and a score of 2 or more was considered to be the presence of OF of the respiratory, cardiovascular, or renal systems^65^. BISAP score was used to assess the severity of pancreatitis. Individual components of the BISAP scoring system were shown in Table 4^68^.

**Table 4:** BISAP scoring system.

BISAP scoring system
Blood urea Nitrogen (BUN) > 25 mg/dL
Impaired mental status (Glasgow Coma Scale Score < 15)
Systemic Inflammatory Response Syndrome (SIRS):
SIRS is defined as presence of two or more of the following
(1) Temperature of < 36 or > 38 °C
(2) Respiratory rate > 20 breaths/min or P a CO2 < 32 mm Hg
(3) Pulse > 90 beats/min
(4) WBC < 4000 or > 12,000 cells/mm 3 or > 10% immature bands
**Age > 60 years**
Pleural effusion detected on imaging

*BISAP* bedside index of severity in acute pancreatitis, *WBC* white blood count.

One point is assigned for each variable within 24 h of presentation and added for a composite score of 0–5.

### Data collection

Age, gender, medical history, and admission number of each patient were collected as baseline demographic data. Moreover, we recorded values of vital signs of all patients on admission and important laboratory parameters. D-dimers were measured in hospital with standard operation procedure; the upper limit of normal value for D-dimer is 0.55 mg/L. Cholesterol (Cho), high-density lipoprotein cholesterol (HDL-C), low-density lipoprotein cholesterol (LDL-C), and triglyceride (TG) were detected in Department of Laboratory of Qilu Hospital. Prothrombin time (PT), activated partial thromboplastin time (APTT), fibrinogen, and thrombin time were also collected. Values of patients who had 2 or more attacks were counted as the average values of every episode. Patients were considered to have dyslipidemia if total cholesterol (Cho) > 240 mg/dL or 6.00 mmol/L, high-density lipoprotein cholesterol (HDL-C) < 40 mg/dL or 0.8 mmol/L, low-density lipoprotein cholesterol (LDL-C) ≥ 160 mg/dL or 3.37 mmol/L, or triglyceride (TG) ≥ 200 mg/dL or 1.7 mmol/L. Coagulation disorder was recorded if an extension of the PT (> 13.8 s) or an extension of the APTT (> 42 s) or D-dimer > 0.55 mg/L occurred. All methods were performed in accordance with the relevant guidelines and regulations.

### Statistic analysis

All statistical analyses were performed using two-tailed test and SPSS version 17.0 and GraphPad Prism v8.0 for Windows (SPSS, Chicago, IL). Data was presented as mean with standard deviation (SD). Comparisons between variables were performed by the one-way ANOVA analysis. For associations between 2 variables, Pearson’s chi-square test or Spearman rank correlation test was applied, as appropriate. The area under the receiver-operating characteristic (ROC) curve (AUC) was used to assess the predictive accuracy of severity of pancreatitis and to determine the optimum cut-off points with optimal sensitivity and specificity. The AUC was calculated using 95% confidence interval (CI). Bivariate logistic regression analysis was adjusted for potential confounding variables, and the odds ratios (OR) and 95% confidence intervals (CI) were calculated. P value less than 0.05 was considered statistically significant.

## Supplementary Information

Supplementary Information.

## Data Availability

All data generated or analysed during this study are included in this published article and its Supplementary Information files.

## Abbreviations

AP: Acute pancreatitis
RAP: Recurrent acute pancreatitis
CP: Chronic pancreatitis
BISAP: Bedside index for severity in acute pancreatitis
AUC: An area under the curve
TG: triglyceride
Cho: Cholesterol
LDL-C: Low-density lipoprotein cholesterol
HDL-C: High-density lipoprotein cholesterol
NEFA: Non-esterified fatty acids
