# Author Correction: Triglyceride to HDL-C ratio is associated with plasma D-dimer levels in different types of pancreatitis

**DOI:** 10.1038/s41598-022-18172-1

**Published:** 2022-08-15

**Authors:** Xiaoqing Jia, Xiaoting Zhang, Dalong Sun, Na Yang, Rong Li, Zheng Luo

**Affiliations:** 1 Department of Gastroenterology and Hepatology, Qilu Hospital of Shandong University, 107 West Wenhua Road, Jinan, 250012 Shandong China; 2 Department of Geriatric Medicine, Qilu Hospital, Shandong University, 107 West Wenhua Road, Jinan, 250012 Shandong China

Correction to: *Scientific Reports* 10.1038/s41598-022-17421-7, published online 28 July 2022

In the original version of this Article Xiaoqing Jia was incorrectly affiliated with ‘Department of Geriatric Medicine, Qilu Hospital, Shandong University, 107 West Wenhua Road, Jinan, 250012, Shandong, China.’ The correct affiliation is listed below.

Department of Gastroenterology and Hepatology, Qilu Hospital of Shandong University, 107 West Wenhua Road, Jinan, 250012, Shandong, China.

The original Article has been corrected.

